# Supplementary material for: Benzodioxane–Benzamides as FtsZ Inhibitors: Effects of Linker’s Functionalization on Gram-Positive Antimicrobial Activity
Source: Antibiotics (Basel). 2023 Dec 8;12(12):1712. doi: 10.3390/antibiotics12121712 (PMC10740499; doi:10.3390/antibiotics12121712)
Supplement: Supplementary file 1 [file antibiotics-12-01712-s001.zip › antibiotics-2682946-supplementary.pdf]

Supporting Information:

Benzodioxane-benzamides as FtsZ inhibitors: effects of linker's functionalization on Gram-positive antimicrobial activity.

Lorenzo Suigo, William Margolin, Eugenia Ulzurrun, Martina Hrast Rambaher, Carlo Zanotto, Victor Sebastián-Pérez, Nuria E. Campillo, Valentina Straniero* and Ermanno Valoti

Summary

***Erythro* 3-[1-(1,4-benzodioxan-2yl)ethyl-1-oxy]-2,6-difluorobenzamide (FZ104)** 2
^{1}H-NMR (DMSO-d_6): 2
^{13}C-NMR (DMSO-d_6): 2
HPLC: 3
***Threo* 3-[1-(1,4-benzodioxan-2yl)ethyl-1-oxy]-2,6-difluorobenzamide (FZ105)** 4
^{1}H-NMR (DMSO-d_6): 4
^{13}C-NMR (DMSO-d6): 4
***Erythro* 3-(2-(1,4-benzodioxan-2-yl)-1-propyloxy)-2,6-difluorobenzamide (FZ98)** 6
^{1}H-NMR (DMSO-d_6): 6
^{13}C-NMR (DMSO-d_6): 6
HPLC: 7
***Threo* 3-(2-(1,4-benzodioxan-2-yl)-1-propyloxy)-2,6-difluorobenzamide (FZ97)** 8
^{1}H-NMR (DMSO-d_6): 8
^{13}C-NMR (DMSO-d_6): 8
HPLC: 9
***Erythro* 3-(2-(5,6,7,8-Tetrahydro-1,4-naphthodioxan-2-yl)prop-1-yloxy)-2,6-difluorobenzamide (FZ118)** 10
^{1}H-NMR (DMSO-d_6): 10
^{13}C-NMR (DMSO-d_6): 10
HPLC: 11
***Threo* 3-(2-(5,6,7,8-Tetrahydro-1,4-naphthodioxan-2-yl)prop-1-yloxy)-2,6-difluorobenzamide (FZ119)** ... 12
^{1}H-NMR (DMSO-d_6): 12
^{13}C-NMR (DMSO-d_6): 12
HPLC: 13
Docking studies 14
Root-Mean-Square Deviation (RMSD) 15
IFD scores 16

Erythro 3-[1-(1,4-benzodioxan-2yl)ethyl-1-oxy]-2,6-difluorobenzamide (FZ104)

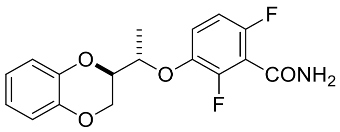

^{1}H-NMR (DMSO-d$_6$):

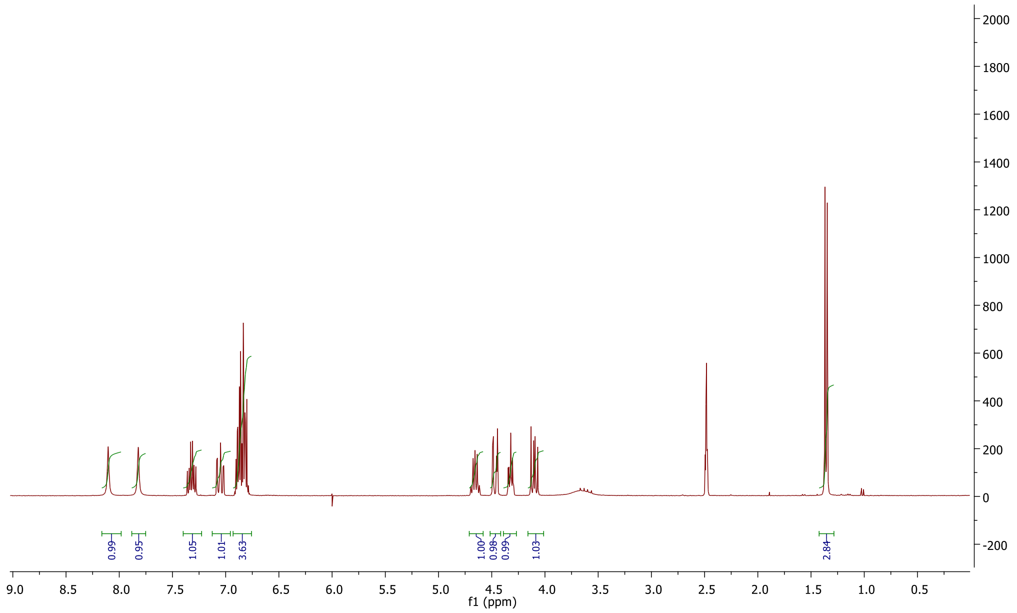

Figure S1: ^{1}H-NMR spectrum of Compound FZ104

^{13}C-NMR (DMSO-d$_6$):

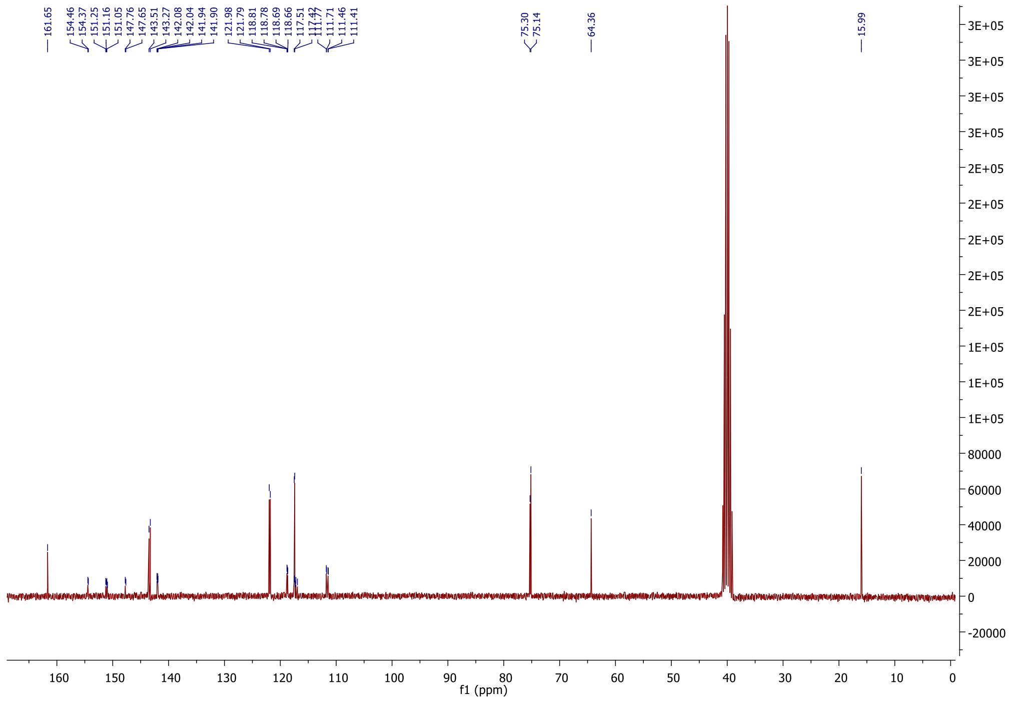

Figure S2: ^{13}C-NMR spectrum of Compound FZ104

HPLC:

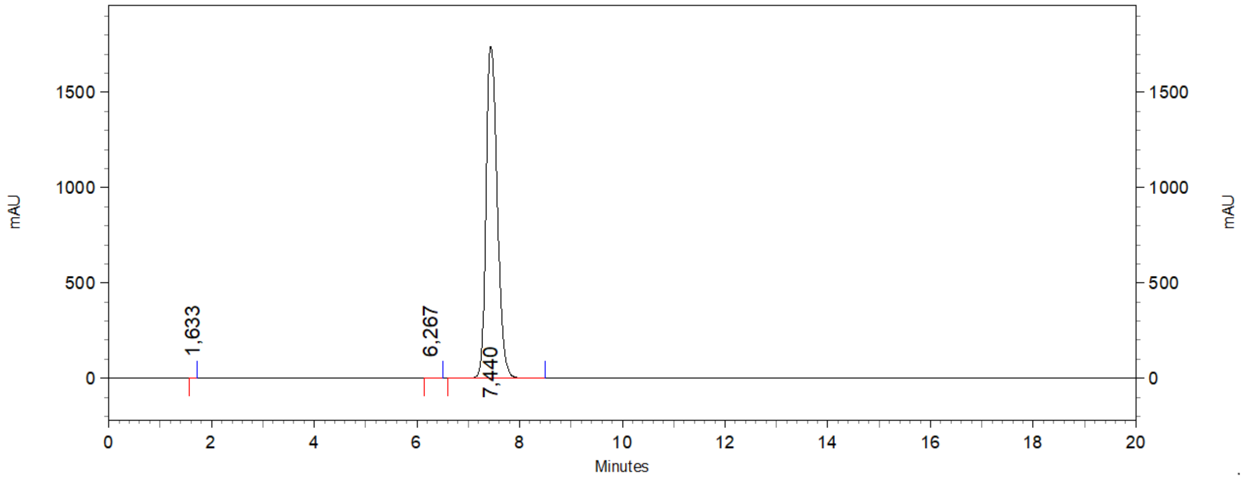

Figure S3: HPLC chromatogram of Compound FZ104

Threo 3-[1-(1,4-benzodioxan-2yl)ethyl-1-oxy]-2,6-difluorobenzamide (FZ105)

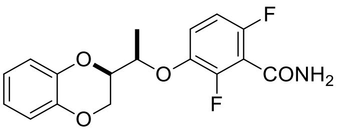

^{1}H-NMR (DMSO-d_6):

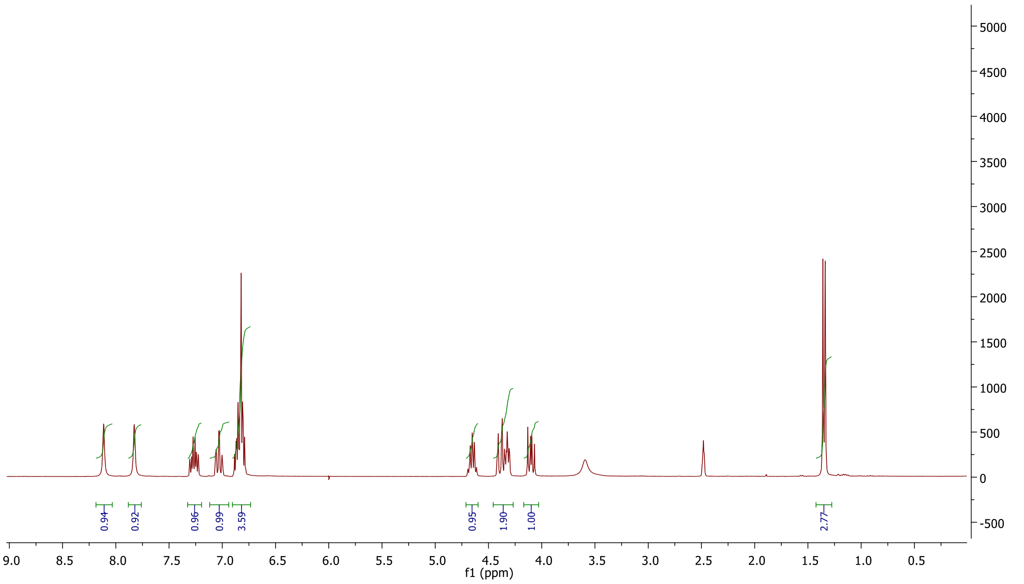

Figure S4: ^{1}H-NMR spectrum of Compound FZ105

^{13}C-NMR (DMSO-d6):

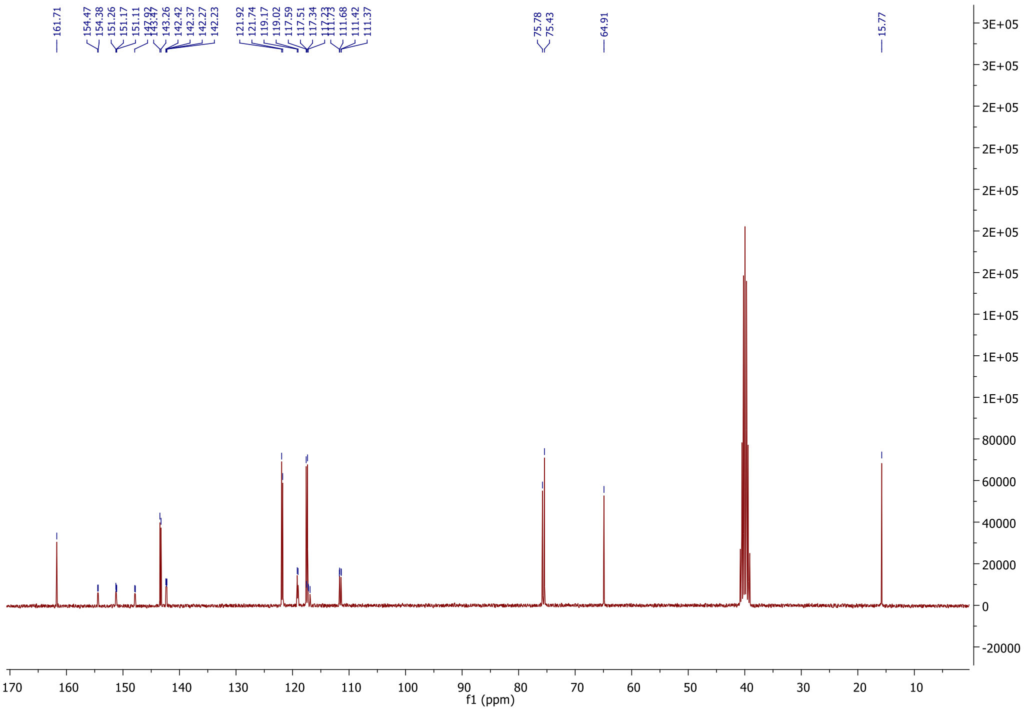

Figure S5: ^{13}C-NMR spectrum of Compound FZ105

HPLC:

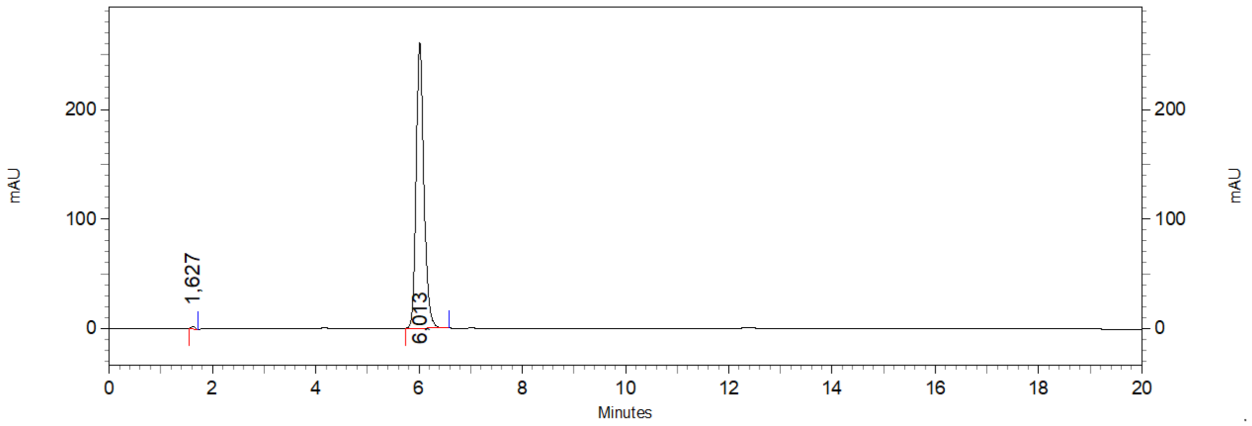

Figure S6: HPLC chromatogram of Compound FZ105

Erythro 3-(2-(1,4-benzodioxan-2-yl)-1-propyloxy)-2,6-difluorobenzamide (FZ98)

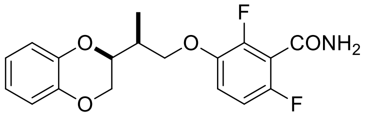

^{1}H-NMR (DMSO-d$_6$):

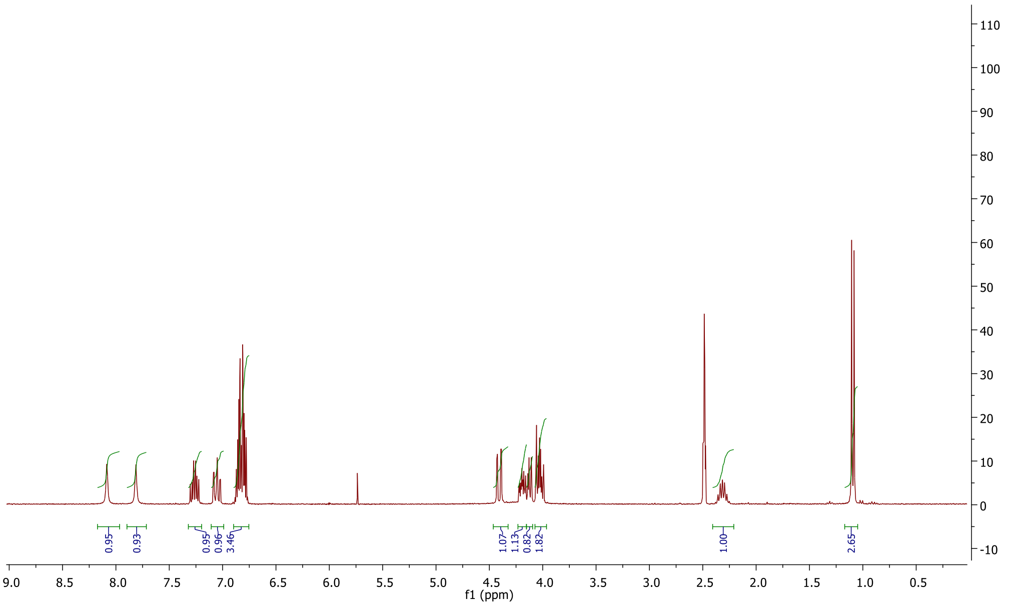

Figure S7: ^{1}H-NMR spectrum of Compound FZ98

^{13}C-NMR (DMSO-d$_6$):

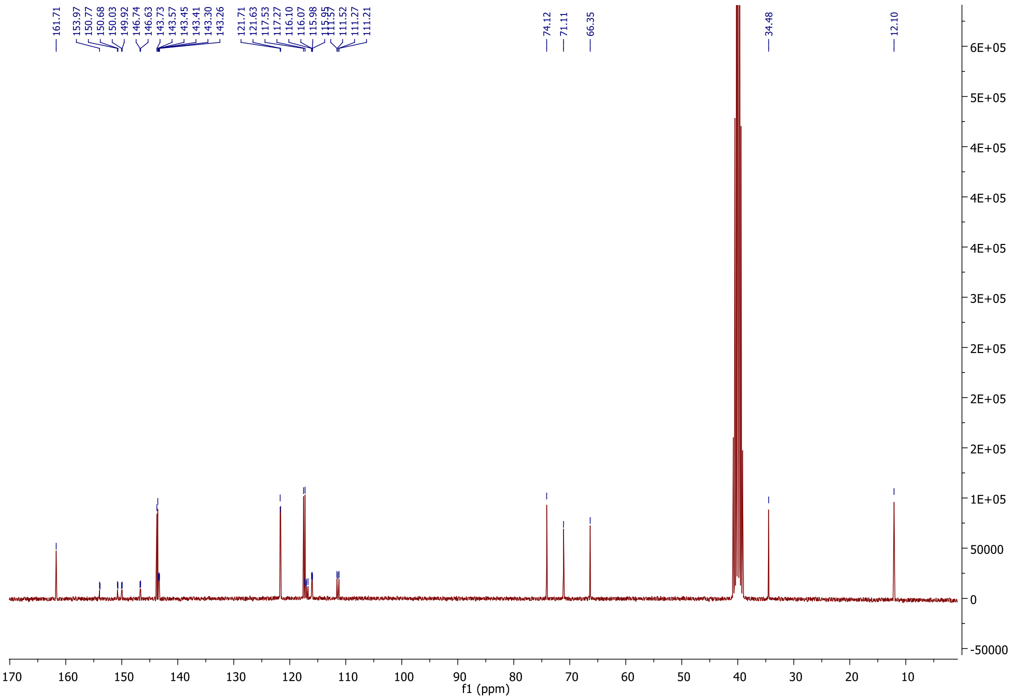

Figure S8: ^{13}C-NMR spectrum of Compound FZ98

HPLC:

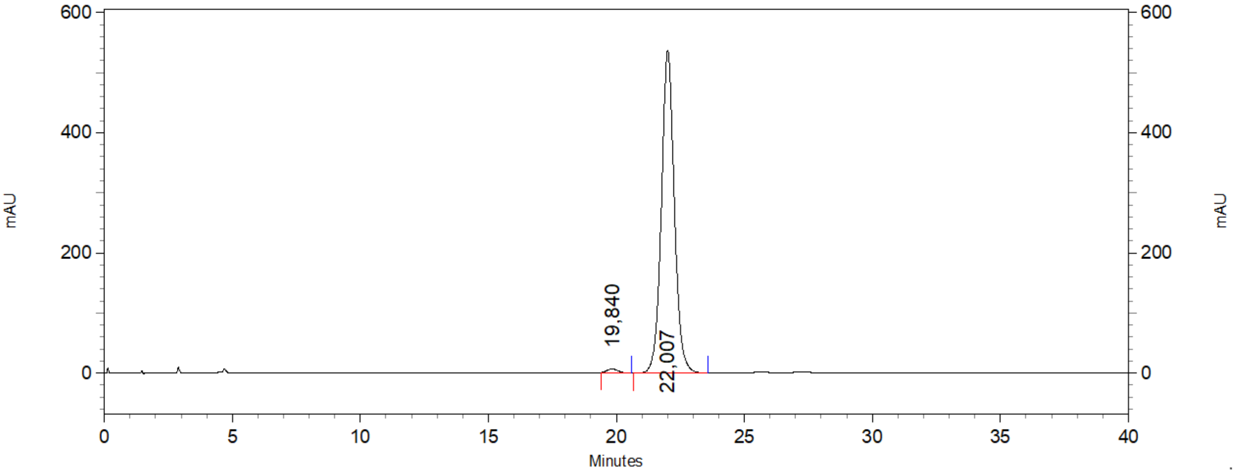

Figure S9: HPLC chromatogram of Compound FZ98

Threo 3-(2-(1,4-benzodioxan-2-yl)-1-propyloxy)-2,6-difluorobenzamide (FZ97)

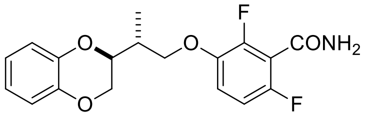

^{1}H-NMR (DMSO-d_6):

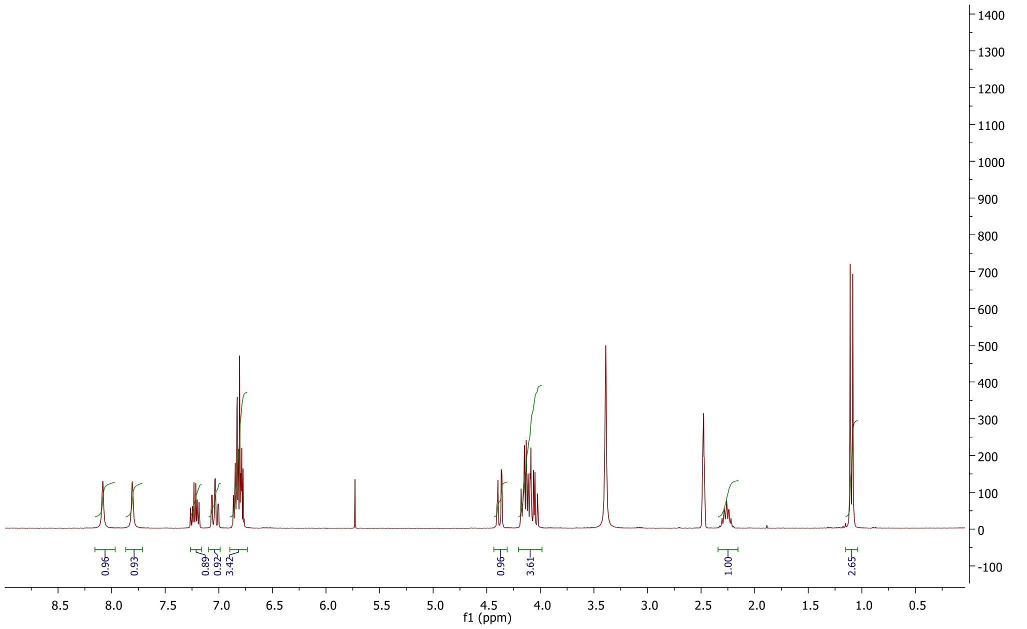

Figure S10: ^{1}H-NMR spectrum of Compound FZ97

^{13}C-NMR (DMSO-d_6):

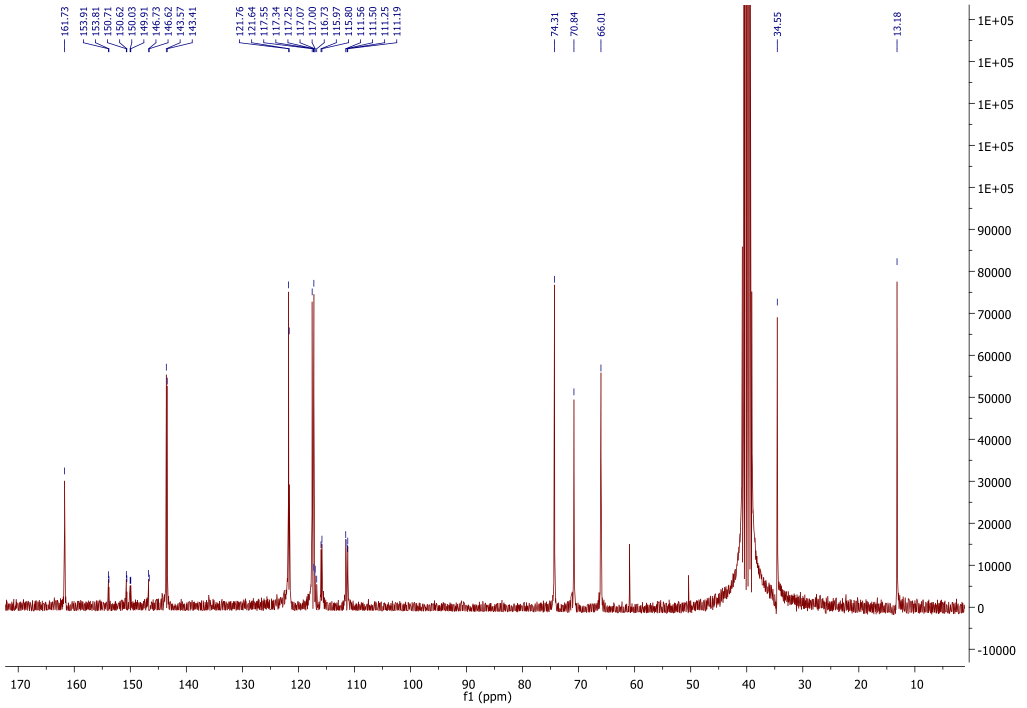

Figure S11: ^{13}C-NMR spectrum of Compound FZ97

HPLC:

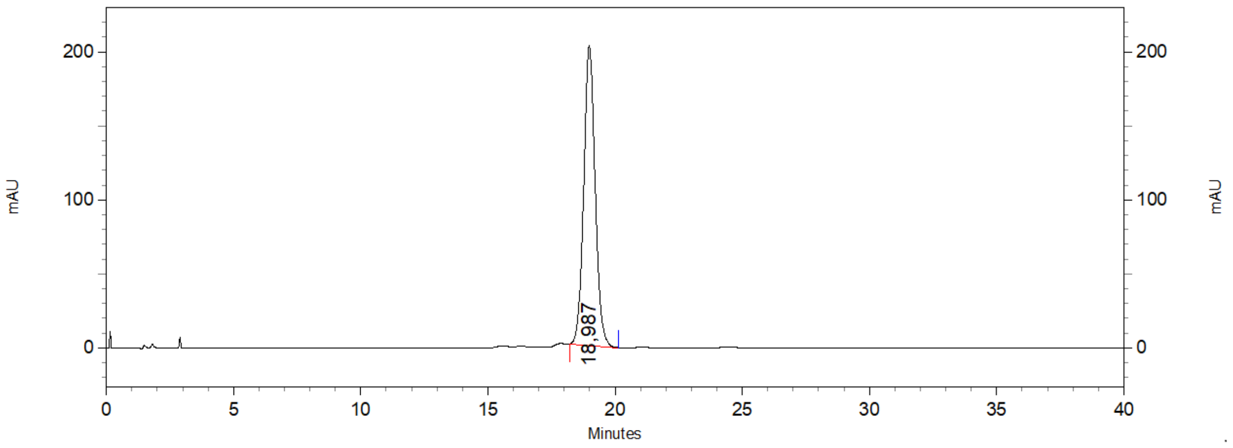

Figure S12: HPLC chromatogram of Compound FZ97

Erythro 3-(2-(5,6,7,8-Tetrahydro-1,4-naphthodioxan-2-yl)prop-1-yloxy)-2,6-difluorobenzamide (FZ118)

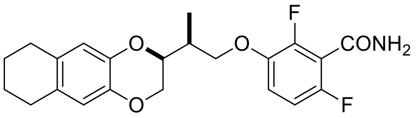

^{1}H-NMR (DMSO-d_6):

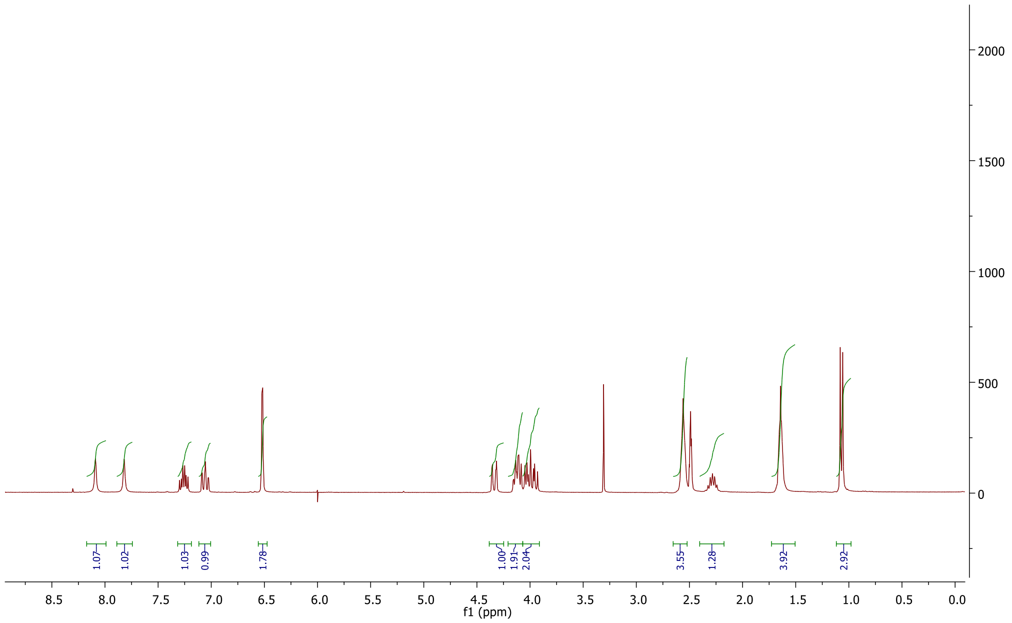

Figure S13: ^{1}H-NMR spectrum of Compound FZ118

^{13}C-NMR (DMSO-d_6):

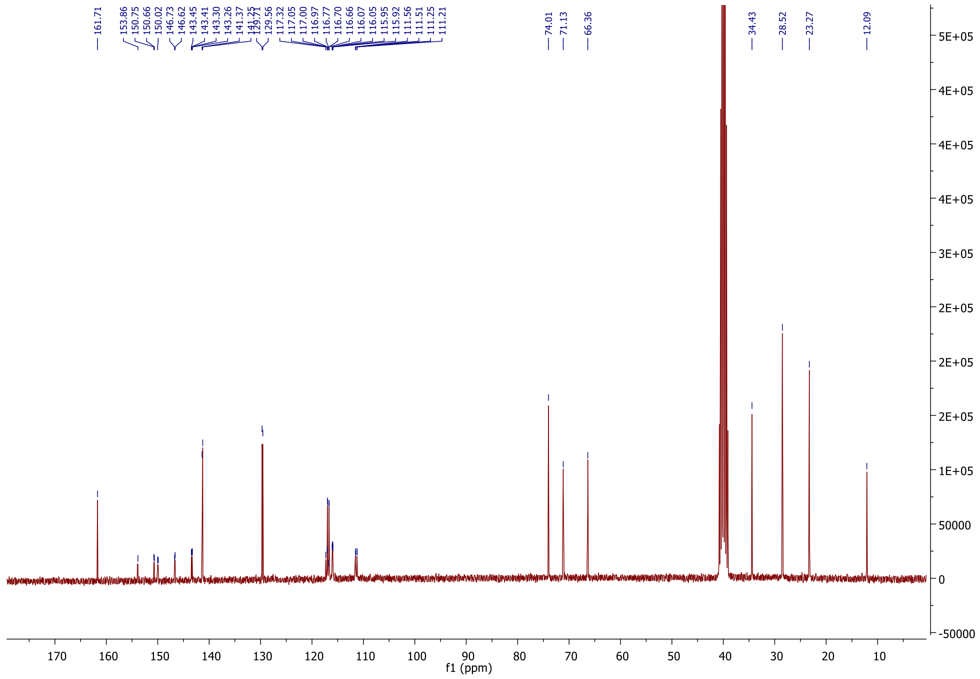

Figure S14: ^{13}C-NMR spectrum of Compound FZ118

HPLC:

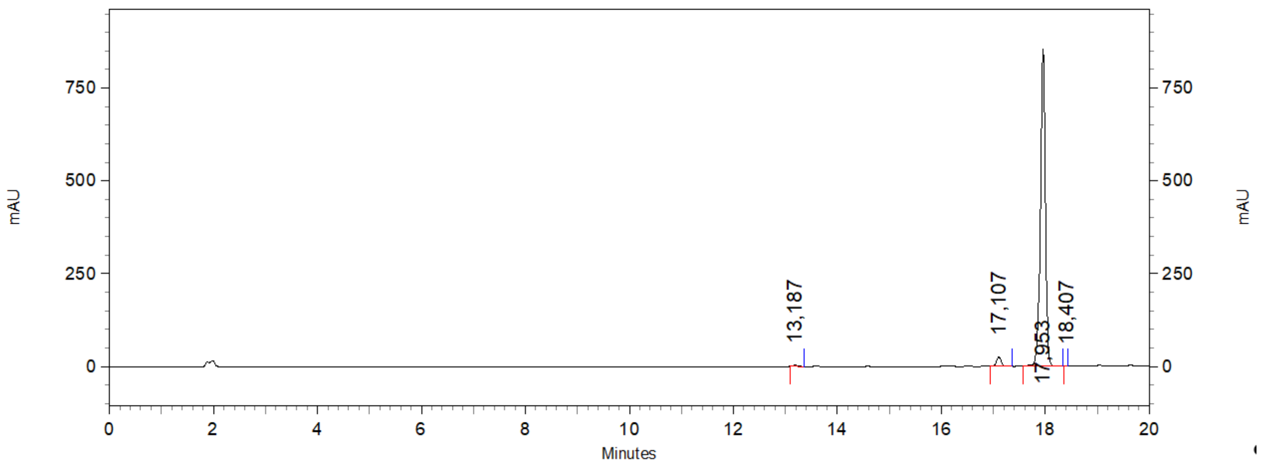

Figure S15: HPLC chromatogram of Compound FZ118

Threo 3-(2-(5,6,7,8-Tetrahydro-1,4-naphthodioxan-2-yl)prop-1-yloxy)-2,6-difluorobenzamide (FZ119)

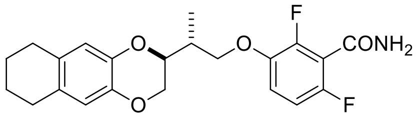

^{1}H-NMR (DMSO-d_6):

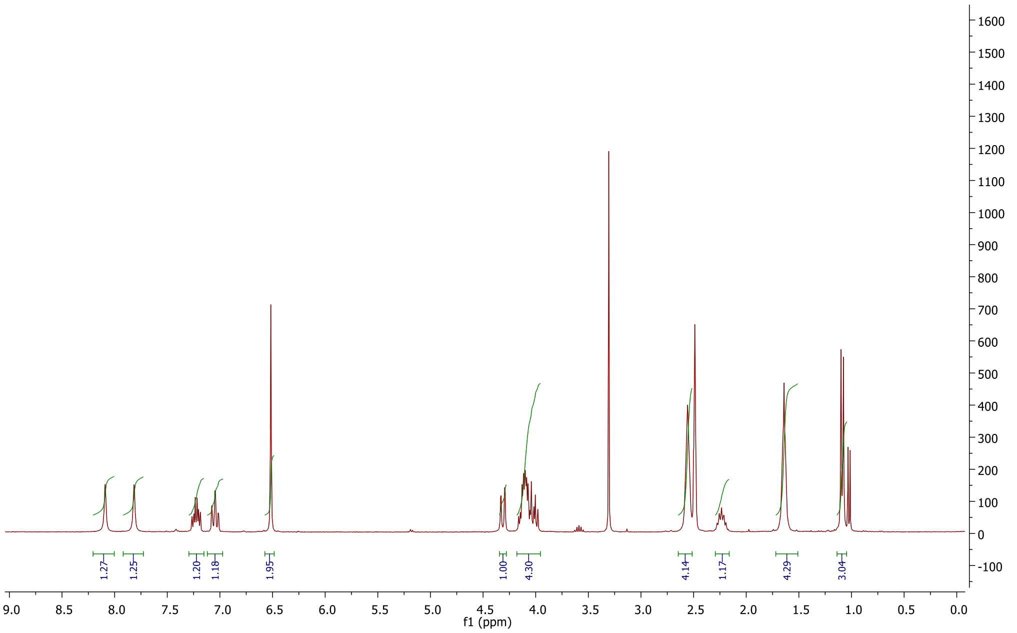

Figure S16: ^{1}H-NMR spectrum of Compound FZ119

^{13}C-NMR (DMSO-d_6):

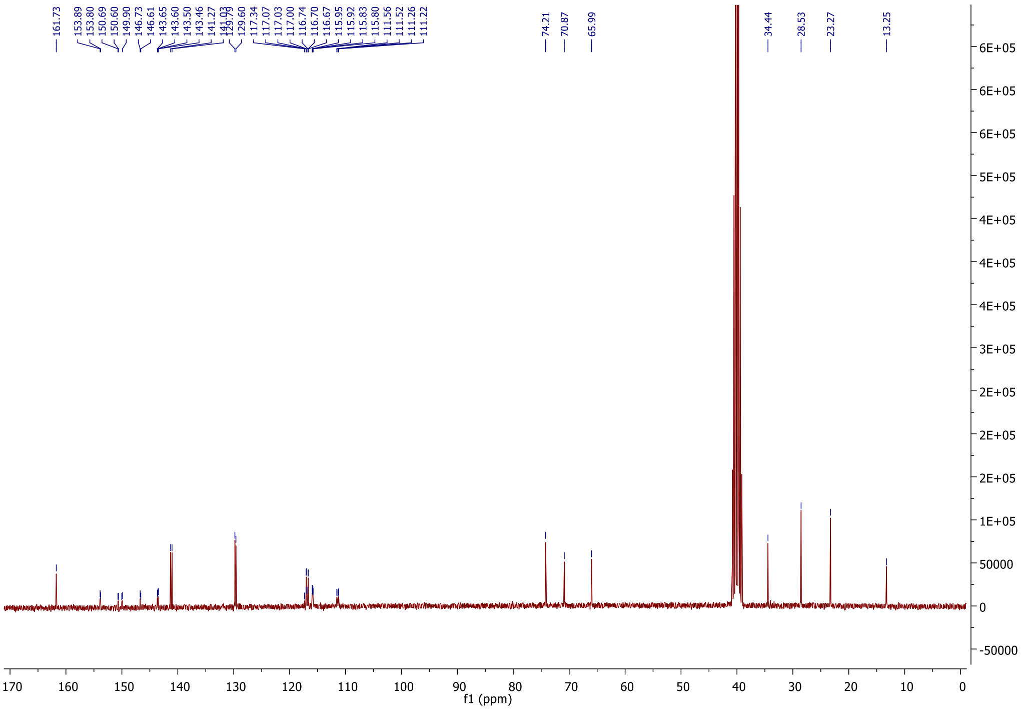

Figure S17: ^{13}C-NMR spectrum of Compound FZ119

HPLC:

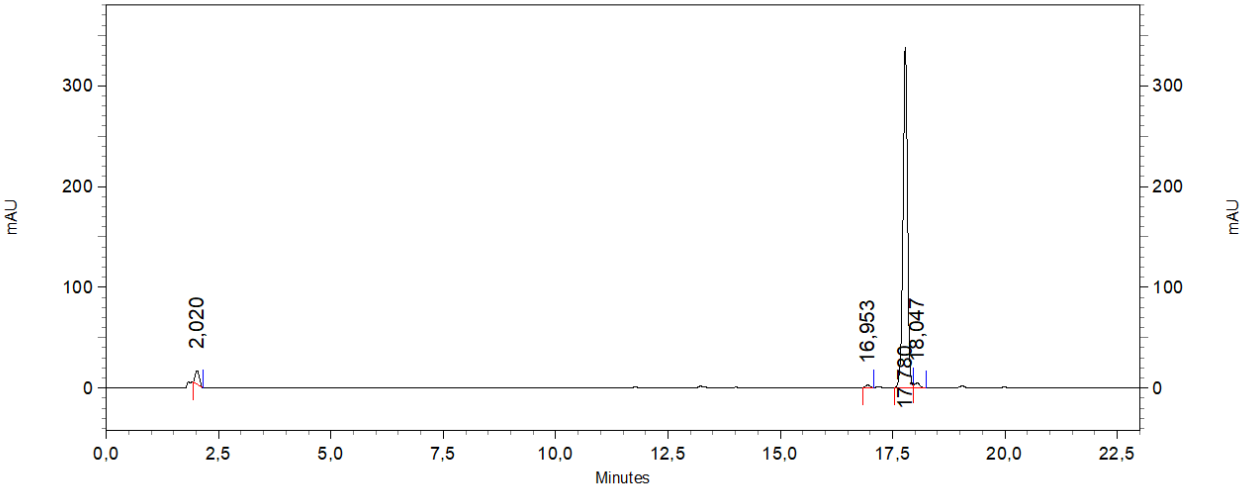

Figure S18: HPLC chromatogram of Compound FZ119

Docking studies

*Table S1: Docking range scores (*kcal/ mol) of the new inhibitors (considering the best and the worst docking poses maintaining the three key hydrogen bonds), correlated to the relative antimicrobial activity towards S. aureus.*

Compound (pose number)	Docking range scores*	MIC *vs S. aureus* (µM)	Compound (pose number)	Docking range scores*	MIC *vs S. aureus* (µM)
**FZ104_*S'R* (4)**	**(-11.468, -8.607)**	**23.9**	**FZ112_*S'R* (14)**	**(-13.364, -10.932)**	**364.4**
**FZ104_*R'S* (5)**	**(-10.935, -9.614)**		**FZ112_*R'S* (15)**	**(-12.904, -9.683)**	
**FZ105_*S'S* (13)**	**(-11.778, -8.269)**	**190.9**	**FZ113_*S'S* (17)**	**(-12.814, -10.783)**	**364.4**
**FZ105_*R'R* (14)**	**(-11.198, -9.519)**		**FZ113_*R'R* (14)**	**(-13.177, -10.887)**	
**FZ97_*R'S* (17)**	**(-11.645, -10.710)**	**366.4**	**FZ116_*S'R* (15)**	**(-14.221, -10.870)**	**4.9**
**FZ97_*S'R* (13)**	**(-11.325, -9.555)**		**FZ116_*R'S* (14)**	**(-14.666, -12.334)**	
**FZ98_*R'R* (11)**	**(-12.295, -10.552)**	**91.6**	**FZ117_*S'S* (13)**	**(-15.470, -11.972)**	**19.7**
**FZ98_*S'S* (16)**	**(-12.754, -10.250)**		**FZ117_*R'R* (12)**	**(-13.790, -11.048)**	
**FZ118_*R'R* (19)**	**(-13.305, -11.611)**	**9.9**			
**FZ118_*S'S* (12)**	**(-15.596, -11.541)**				
**FZ119_*R'S* (14)**	**(-12.803, -11.386)**	**9.9**			
**FZ119_*S'R* (16)**	**(-14.200, -11.170)**				

Root-Mean-Square Deviation (RMSD)

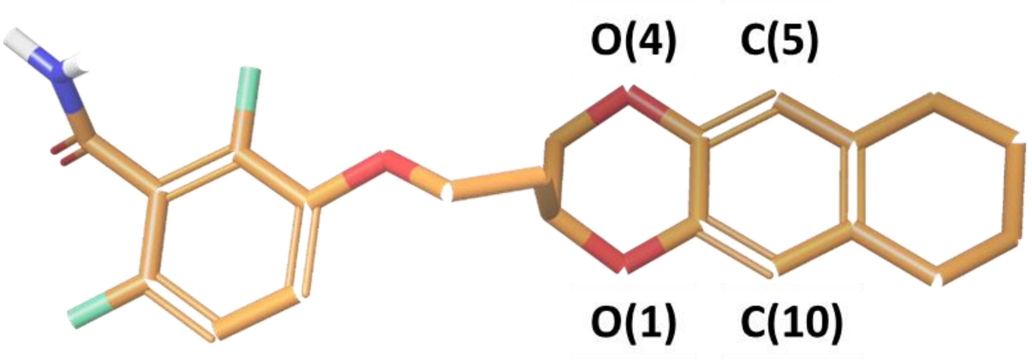

Figure S19: Legenda for O(1), O(4), C(5) and C(10) labeling, to calculate the RMSD in the superposition FZ100-novel compounds

Table S2: RMSD for atom pairs between O(1), O(4), C(5) and C(10) using FZ100 as reference structure

**Inhibitor Pose**	**RMSD O(4) (Å)**	**RMSD O(1) (Å)**	**RMSD C(5) (Å)**	**RMSD C(10) (Å)**	**180º rotation**
1_FZ104_*R'S*	0.883	0.865	0.773	0.807	not
2_FZ116_*R'S*	0.515	0.148	0.572	0.256	yes
7_FZ117_*S'S*	0.581	0.606	0.809	0.881	yes
10_FZ117_*S'S*	0.744	0.917	0.36	0.847	yes
2_FZ118_*R'R*	0.169	0.129	0.816	0.941	yes
3_FZ119_*R'S*	0.332	0.384	0.530	0.557	yes
4_FZ119_*R'S*	0.400	0.305	0.514	0.668	yes

IFD scores

Table S3

Title	docking score
1_FZ104_R’S	-10.935
1_FZ104_R’S	-10.988
1_FZ104_R’S	-10.985
1_FZ104_R’S	-10.294
1_FZ104_R’S	-9.614
1_FZ104_S’R	-11.468
1_FZ104_S’R	-10.571
1_FZ104_S’R	-9.293
1_FZ104_S’R	-8.607
2_FZ105_R’R	-11.198
2_FZ105_R’R	-11.378
2_FZ105_R’R	-10.998
2_FZ105_R’R	-10.72
2_FZ105_R’R	-10.701
2_FZ105_R’R	-11.009
2_FZ105_R’R	-11.298
2_FZ105_R’R	-10.548
2_FZ105_R’R	-10.42
2_FZ105_R’R	-10.59
2_FZ105_R’R	-10.78
2_FZ105_R’R	-10.661
2_FZ105_R’R	-10.172
2_FZ105_R’R	-9.519
2_FZ105_S’S	-11.778
2_FZ105_S’S	-11.254
2_FZ105_S’S	-10.863
2_FZ105_S’S	-10.693
2_FZ105_S’S	-10.352
2_FZ105_S’S	-11.348
2_FZ105_S’S	-10.381
2_FZ105_S’S	-9.818
2_FZ105_S’S	-10.122
2_FZ105_S’S	-9.208
2_FZ105_S’S	-9.951
2_FZ105_S’S	-9.315
2_FZ105_S’S	-8.269
3_FZ97_R’S	-11.645
3_FZ97_R’S	-11.446
3_FZ97_R’S	-11.194
3_FZ97_R’S	-11.631
3_FZ97_R’S	-10.775
3_FZ97_R’S	-11.139
3_FZ97_R’S	-11.326
3_FZ97_R’S	-10.719
3_FZ97_R’S	-11.063
3_FZ97_R’S	-11.552
3_FZ97_R’S	-11.298
3_FZ97_R’S	-11.347
3_FZ97_R’S	-10.87
3_FZ97_R’S	-10.924
3_FZ97_R’S	-10.945
3_FZ97_R’S	-10.185
3_FZ97_R’S	-10.71
3_FZ97_S’R	-11.325
3_FZ97_S’R	-11.25
3_FZ97_S’R	-10.745
3_FZ97_S’R	-11.164
3_FZ97_S’R	-10.889
3_FZ97_S’R	-11.762
3_FZ97_S’R	-10.88
3_FZ97_S’R	-11.236
3_FZ97_S’R	-11.125
3_FZ97_S’R	-11.136
3_FZ97_S’R	-11.039
3_FZ97_S’R	-10.823
3_FZ97_S’R	-9.555
4_FZ98_R’R	-12.295
4_FZ98_R’R	-12.051
4_FZ98_R’R	-12.181
4_FZ98_R’R	-12.072
4_FZ98_R’R	-11.59
4_FZ98_R’R	-11.63
4_FZ98_R’R	-11.021
4_FZ98_R’R	-11.307
4_FZ98_R’R	-11.022
4_FZ98_R’R	-10.863
4_FZ98_R’R	-10.552
4_FZ98_S’S	-12.754
4_FZ98_S’S	-11.747
4_FZ98_S’S	-11.661
4_FZ98_S’S	-11.655
4_FZ98_S’S	-12.197
4_FZ98_S’S	-12
4_FZ98_S’S	-10.641
4_FZ98_S’S	-11.39
4_FZ98_S’S	-11.01
4_FZ98_S’S	-11.05
4_FZ98_S’S	-10.705
4_FZ98_S’S	-10.632
4_FZ98_S’S	-10.445
4_FZ98_S’S	-10.767
4_FZ98_S’S	-10.577
4_FZ98_S’S	-10.25
5_FZ112_R’S	-12.904
5_FZ112_R’S	-13.132
5_FZ112_R’S	-12.821
5_FZ112_R’S	-12.788
5_FZ112_R’S	-12.105
5_FZ112_R’S	-12.268
5_FZ112_R’S	-11.986
5_FZ112_R’S	-12.135
5_FZ112_R’S	-11.681
5_FZ112_R’S	-11.826
5_FZ112_R’S	-11.057
5_FZ112_R’S	-10.773
5_FZ112_R’S	-10.39
5_FZ112_R’S	-10.312
5_FZ112_R’S	-9.683
5_FZ112_S’R	-13.364
5_FZ112_S’R	-13.738
5_FZ112_S’R	-12.656
5_FZ112_S’R	-12.474
5_FZ112_S’R	-13.215
5_FZ112_S’R	-11.885
5_FZ112_S’R	-12.884
5_FZ112_S’R	-12.383
5_FZ112_S’R	-11.897
5_FZ112_S’R	-11.879
5_FZ112_S’R	-11.073
5_FZ112_S’R	-11.236
5_FZ112_S’R	-10.903
5_FZ112_S’R	-10.932
6_FZ113_R’R	-13.177
6_FZ113_R’R	-13.212
6_FZ113_R’R	-12.148
6_FZ113_R’R	-12.877
6_FZ113_R’R	-13.413
6_FZ113_R’R	-12.544
6_FZ113_R’R	-12.31
6_FZ113_R’R	-12
6_FZ113_R’R	-11.694
6_FZ113_R’R	-11.35
6_FZ113_R’R	-11.336
6_FZ113_R’R	-11.092
6_FZ113_R’R	-10.675
6_FZ113_R’R	-10.887
6_FZ113_S’S	-12.814
6_FZ113_S’S	-11.957

6_FZ113_S’S	-11.803
6_FZ113_S’S	-13.147
6_FZ113_S’S	-12.217
6_FZ113_S’S	-11.752
6_FZ113_S’S	-11.621
6_FZ113_S’S	-11.8
6_FZ113_S’S	-11.749
6_FZ113_S’S	-11.598
6_FZ113_S’S	-11.583
6_FZ113_S’S	-11.634
6_FZ113_S’S	-11.393
6_FZ113_S’S	-11.551
6_FZ113_S’S	-11.406
6_FZ113_S’S	-11.501
6_FZ113_S’S	-10.783
7_FZ116_R’S	-14.666
7_FZ116_R’S	-14.631
7_FZ116_R’S	-14.165
7_FZ116_R’S	-13.899
7_FZ116_R’S	-13.926
7_FZ116_R’S	-12.833
7_FZ116_R’S	-13.598
7_FZ116_R’S	-13.678
7_FZ116_R’S	-13.823
7_FZ116_R’S	-13.366
7_FZ116_R’S	-12.694
7_FZ116_R’S	-12.896
7_FZ116_R’S	-13.312
7_FZ116_R’S	-12.334
7_FZ116_S’R	-14.221
7_FZ116_S’R	-13.397
7_FZ116_S’R	-13.12
7_FZ116_S’R	-13.38
7_FZ116_S’R	-13.506
7_FZ116_S’R	-13.383
7_FZ116_S’R	-12.29
7_FZ116_S’R	-12.791
7_FZ116_S’R	-13.289
7_FZ116_S’R	-12.848
7_FZ116_S’R	-12.773
7_FZ116_S’R	-12.308
7_FZ116_S’R	-11.716
7_FZ116_S’R	-11.338
7_FZ116_S’R	-10.87
8_FZ117_R’R	-13.79
8_FZ117_R’R	-13.941
8_FZ117_R’R	-13.863
8_FZ117_R’R	-14.278
8_FZ117_R’R	-14.045
8_FZ117_R’R	-13.667
8_FZ117_R’R	-13.425
8_FZ117_R’R	-13.471
8_FZ117_R’R	-13.021
8_FZ117_R’R	-12.784
8_FZ117_R’R	-12.525
8_FZ117_R’R	-11.048
8_FZ117_S’S	-15.47
8_FZ117_S’S	-14.841
8_FZ117_S’S	-14.968
8_FZ117_S’S	-13.518
8_FZ117_S’S	-13.303
8_FZ117_S’S	-13.605
8_FZ117_S’S	-13.438
8_FZ117_S’S	-13.559
8_FZ117_S’S	-12.733
8_FZ117_S’S	-12.537
8_FZ117_S’S	-12.415
8_FZ117_S’S	-12.191
8_FZ117_S’S	-11.972
9_FZ118_R’R	-13.305
9_FZ118_R’R	-12.865
9_FZ118_R’R	-13.127
9_FZ118_R’R	-13.32
9_FZ118_R’R	-13.584
9_FZ118_R’R	-12.741
9_FZ118_R’R	-13.254
9_FZ118_R’R	-12.9
9_FZ118_R’R	-13.026
9_FZ118_R’R	-12.777
9_FZ118_R’R	-12.697
9_FZ118_R’R	-11.861
9_FZ118_R’R	-12.418
9_FZ118_R’R	-12.093
9_FZ118_R’R	-12.328
9_FZ118_R’R	-11.838
9_FZ118_R’R	-12.286
9_FZ118_R’R	-11.572
9_FZ118_R’R	-11.611
9_FZ118_S’S	-15.596
9_FZ118_S’S	-13.581
9_FZ118_S’S	-14.049
9_FZ118_S’S	-13.534
9_FZ118_S’S	-13.213
9_FZ118_S’S	-12.668
9_FZ118_S’S	-12.475
9_FZ118_S’S	-12.303
9_FZ118_S’S	-12.371
9_FZ118_S’S	-12.316
9_FZ118_S’S	-11.804
9_FZ118_S’S	-11.541
10_FZ119_R’S	-12.803
10_FZ119_R’S	-12.656
10_FZ119_R’S	-13.023
10_FZ119_R’S	-12.869
10_FZ119_R’S	-13.176
10_FZ119_R’S	-12.827
10_FZ119_R’S	-12.429
10_FZ119_R’S	-12.698
10_FZ119_R’S	-12.44
10_FZ119_R’S	-12.558
10_FZ119_R’S	-12.27
10_FZ119_R’S	-12.517
10_FZ119_R’S	-11.913
10_FZ119_R’S	-11.386
10_FZ119_S’R	-14.2
10_FZ119_S’R	-13.369
10_FZ119_S’R	-13.256
10_FZ119_S’R	-13.533
10_FZ119_S’R	-13.357
10_FZ119_S’R	-13.32
10_FZ119_S’R	-13.396
10_FZ119_S’R	-13.411
10_FZ119_S’R	-13.012
10_FZ119_S’R	-12.475
10_FZ119_S’R	-12.478
10_FZ119_S’R	-12.541
10_FZ119_S’R	-12.885
10_FZ119_S’R	-12.368
10_FZ119_S’R	-12.87
10_FZ119_S’R	-11.17
